# Congenital Cardiac Outflow Tract Abnormalities in Dogs: Prevalence and Pattern of Inheritance From 2008 to 2017

**DOI:** 10.3389/fvets.2019.00052

**Published:** 2019-02-27

**Authors:** Eric S. Ontiveros, Samantha L. Fousse, Amanda E. Crofton, Timothy E. Hodge, Catherine T. Gunther-Harrington, Lance C. Visser, Joshua A. Stern

**Affiliations:** ^1^Department of Medicine and Epidemiology, School of Veterinary Medicine, University of California, Davis, Davis, CA, United States; ^2^University of California Veterinary Medical Center, San Diego, CA, United States

**Keywords:** genetics, subvalvular aortic stenosis, pulmonic stenosis, congenital heart disease, prevalence, inheritance, veterinary

## Abstract

Subvalvular aortic stenosis (SAS) and valvular pulmonic stenosis (PS) are two of the most common congenital heart diseases of dogs. The aim of this study was to determine the prevalence and mode of inheritance of these congenital heart diseases in a large veterinary teaching hospital population. Case records of dogs presented to the University of California Davis, Veterinary Medical Teaching Hospital (UCD VMTH) between January 2008 to December 2017 were reviewed retrospectively and pedigree information was obtained when available. There were 259 unique SAS and 336 unique PS cases diagnosed during the study period. The prevalence of SAS was 0.3% of overall hospital admissions and 4.7% for all dogs seen by the cardiology service. The prevalence for PS was 0.41% of overall hospital admissions and 6.1% of dogs seen by the cardiology service. Bullmastiffs and Newfoundlands had the greatest prevalence (6.59 and 4.46%, respectively) and odds ratio (52.43 and 34.73, respectively) for SAS. Bulldogs and French Bulldogs had the greatest prevalence (4.8 and 2.7%, respectively) and odds ratio (13.32 and 7.52, respectively) for PS. The identified prevalence of SAS and PS is higher than previously reported. Pedigree analysis in SAS affected Bullmastiffs, Golden Retrievers, and Rottweilers suggested an autosomal recessive pattern of inheritance. The mode of inheritance for PS in Bulldogs, also appears to be autosomal recessive. The results of this study can be used to inform future selection of breeding pairs and genetic studies aimed at reducing the prevalence of these common congenital heart diseases.

## Introduction

**Figure 1:**
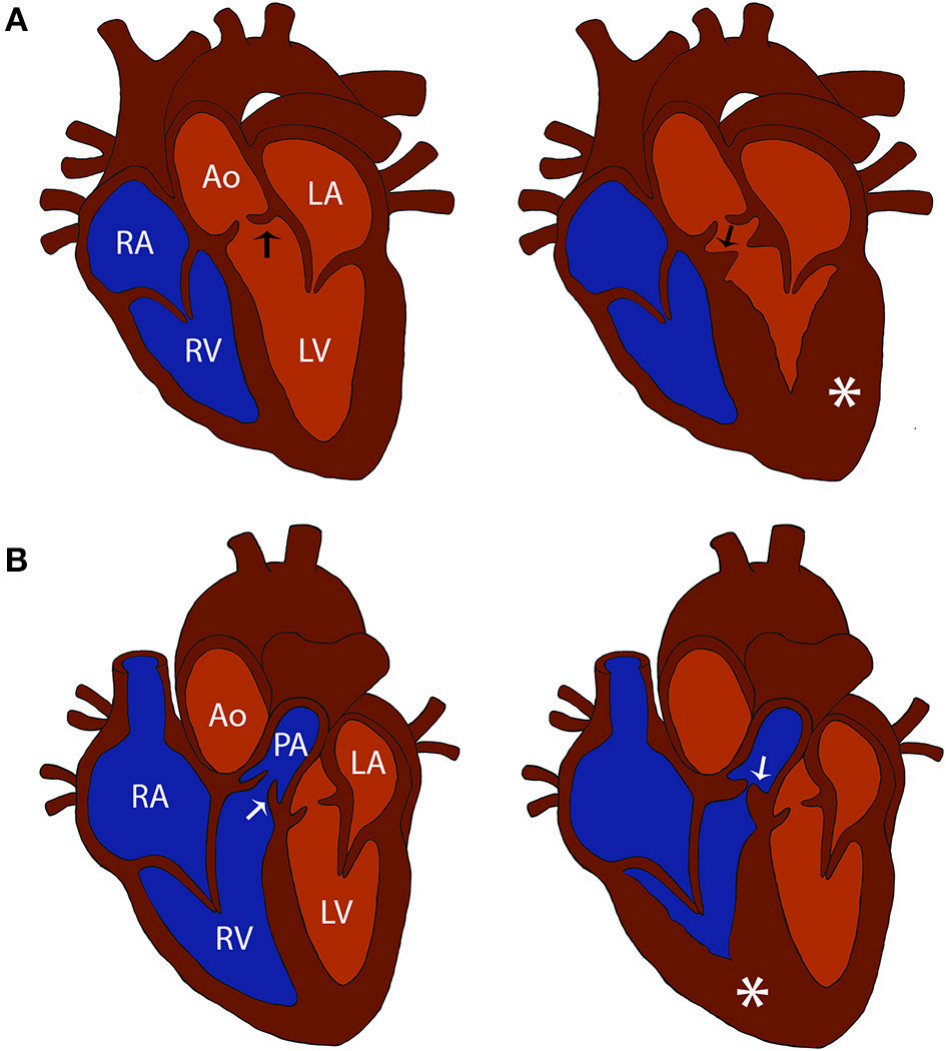
Comparison of normal and cardiac outflow tract abnormalities affected hearts. **(A)** Diagrams of a normal heart and a heart affected with subvalvular aortic stenosis (SAS). Note the stenosis caused by the subvalvular ridge (arrow) and left ventricular hypertrophy (asterisk) in the SAS affected heart compared to the normal heart. **(B)** Diagrams of a normal heart and a heart affected with pulmonic stenosis (PS). Note the stenosis caused by thickened pulmonic valve leaflets (arrow) and right ventricular hypertrophy (asterisk) in the PS affected heart compared to the normal heart. Structures in the normal heart diagrams are labeled for reference: RV, right ventricle; RA, right atrium; LV, left ventricle; LA, left atrium; Ao, aorta; PA, pulmonary artery; black arrow, aortic valve; white arrow, pulmonic valve.

Subvalvular aortic stenosis (SAS) and valvular pulmonic stenosis (PS) are two of the most common congenital heart diseases diagnosed in dogs (1, 2). Both diseases involve stenosis of a cardiac outflow tract that results in pressure overload to the respective ventricle and may lead to adverse clinical outcomes such as congestive heart failure or sudden cardiac death (Figure 1). It was previously reported that certain dog breeds have a higher relative risk for developing congenital heart disease. The breeds with a reported predisposition to SAS include Boxers, German Shepherds, Dogue de Bordeaux, Newfoundlands, Rottweilers, and Golden Retrievers (2). The breeds with a reported predisposition to PS include Boxers, Bulldogs, and French Bulldogs (2). Breed specific disease predisposition suggests an underlying genetic etiology for these conditions (3).

The gold standard for diagnosing SAS is via cardiac necropsy, in which the presence of fibromuscular nodules, a ridge, or ring is observed below the aortic valve (4). For valvular PS cases, commissural fusion of the valve leaflets, severe thickening of the valve leaflets, and/or annular hypoplasia may be seen at necropsy (5). For ante-mortem diagnostics, a veterinary cardiologist can perform an echocardiogram exam to assess whether a dog is affected with SAS or PS. A standard echocardiogram assessment used to determine SAS or PS disease status includes 2D, M-mode, color-flow Doppler, and continuous-wave Doppler modalities which allow determination of severity and morphology of the stenotic lesion (6).

Prognosis for SAS and PS varies depending on severity and treatment availability. Dogs diagnosed with moderate or severe SAS have an average lifespan of 19 months without medication or intervention (7). Moderately and severely affected SAS cases can develop cardiac complications that result in sudden death, infective endocarditis, and/or congestive heart failure (7). Currently, the only medical treatment available for dogs affected with SAS is administration of beta-adrenoreceptor blocking drugs. Severely affected SAS cases treated with beta-adrenoreceptor blocking drugs were shown to have a median survival time of 56 months (8). Although balloon valvuloplasty is an option for SAS affected cases, it has been demonstrated that it does not significantly increase life expectancy when compared to beta-adrenoreceptor blockers alone (8).

Of dogs diagnosed with severe PS, one study found that 53% die before 1 year of age without intervention (9). Available treatments for PS include administration of beta-adrenoreceptor blocking drugs and percutaneous pulmonary balloon valvuloplasty (9). When treated with percutaneous pulmonary balloon valvuloplasty, only 4% of severe cases were shown to die before 1 year of age (9). However, not all PS cases can benefit from a percutaneous pulmonary balloon valvuloplasty. For example, dogs with dysplastic valves and pulmonic annulus hypoplasia have a worse post-valvuloplasty outcome compared to those with fused valves and a normal annulus diameter (10). Additionally, if an aberrant prepulmonic coronary artery is identified, a traditional balloon valvuloplasty is considered to be contraindicated (11). The breeds with a reported predisposition to coronary artery anomalies include Bulldogs and Boxers (12).

Both SAS and PS are heritable congenital heart diseases affecting the cardiac outflow tracts of humans and dogs (3, 13, 14). Previous studies have suggested that SAS can have an autosomal dominant with incomplete penetrance or polygenic mode of inheritance in humans and the Newfoundland breed (4, 14, 15). In Golden Retrievers, an inconclusive pedigree analysis suggested either a recessive or polygenic mode of inheritance for SAS (16). To date, the only identified variant associated with canine SAS is an insertion in the *PICALM* gene of Newfoundlands (14). Although the variant identified is highly penetrant at 80.6% in the Newfoundland breed, the manuscript illustrates that it does not fulfill the criteria of a sole causal variant. Thus, additional genetic studies to identify variants are warranted.

In humans, congenital valvular PS presents in syndromic and non-syndromic forms with varying modes of inheritance (17). Mutations involving the Ras signaling pathway are associated with many of the syndromic forms of PS, while only *GATA4* has been associated with the non-syndromic form (17). Although, some genetic risk variants have been associated with human PS, for many children the causative variant remains unknown (17, 18). An extensive genetic study in Beagles with PS suggested that the disease is polygenic and inherited in a recessive manner (5). However, there is no conclusive evidence regarding the inheritance of PS in commonly affected breeds, which hinders breeding efforts to reduce disease prevalence in affected breeds. To date there are no reported variants associated with the development of canine PS.

It has been long understood that specific breeds have increased risk for developing SAS and/or PS, however information on the mode of inheritance in these breeds is limited (7). The aim of our study is to determine the prevalence of these congenital heart diseases in a large veterinary teaching hospital. Furthermore, we aim to elucidate the mode of inheritance for PS/SAS in breeds where pedigree information on a large number of cases is available. It is essential to assess prevalence and inheritance to aid researchers in developing novel therapeutic approaches or genetic tests that can be used to decrease the prevalence of these conditions.

## Materials and Methods

### Sample Population and Phenotyping

Case records of dogs presented to the University of California Davis, Veterinary Medical Teaching Hospital (UCD VMTH) between January 2008 to December 2017 were reviewed retrospectively. Only cases diagnosed echocardiographically with SAS or PS by a board-certified cardiologist or cardiology resident under the direct supervision of a cardiologist at the UCD VMTH were included. Hospital records were reviewed to obtain information on the breed, sex, and selected echocardiographic findings used for diagnosis. Additionally, the presence and absence of coronary anomalies diagnosed by coronary angiography in PS dogs was recorded if available. Exclusion criteria included dogs with acquired stenosis or other structural congenital heart defects that could influence the accuracy of obtaining outflow tract velocities.

Evaluation of the stenotic lesion with color and continuous wave Doppler along with standard 2D and M-mode echocardiographic measurements were completed according to previously published studies (6, 19). Diagnosis of SAS required measured elevated aortic outflow velocities (AoV) obtained from the subcostal view by continuous-wave spectral Doppler imaging (6). The subvalvular nature of the stenosis was documented by either clear 2D evidence of a subvalvular ridge/ring in the LVOT and/or a subvalvular step-up gradient identified by spectral pulsed-wave Doppler. Briefly, PS was diagnosed using the right parasternal short axis view by visualizing abnormal pulmonary valve morphology and elevated pulmonic outflow velocities (PoV) obtained by continuous-wave spectral Doppler imaging (6).

Dogs were classified into severity categories (equivocal, mild, moderate, and severe) based on their peak modal recorded AoV or peak modal recorded PoV. Dogs affected with SAS were categorized as equivocal (AoV: 2.0–2.5 m/s), mild (AoV: 2.5–3.5 m/s), moderate (AoV: 3.5–4.5 m/s), or severe (AoV: >4.5 m/s). Similarly, severity of PS was based on PoV and categorized as equivocal (PoV: 1.5–2.5 m/s), mild (PoV: 2.5–3.5 m/s), moderate (PoV: 3.5–4.5 m/s), and severe (PoV: >4.5 m/s). The mild, moderate, and severe categories for both SAS and PS were based off previous literature (1, 6), however a conservative equivocal range was used to reduce misclassification of cases and controls.

### Prevalence Calculation

The prevalence of SAS and PS were calculated overall for the hospital's canine population and by breed. Dogs reported to be equivocal for SAS or PS were excluded from this analysis since their phenotype cannot be definitively described. Inclusion of equivocal dogs can result in an overestimated prevalence if combined with the affected dogs or an underestimated prevalence if combined with the control dogs.

All statistics were completed using GraphPad Prism Software v7 (La Jolla, CA). Odds ratios, 95% confidence intervals, and chi-square *p*-values were calculated to determine if a breed or sex predisposition was present for SAS or PS breeds with at least 10 reported cases during the study period. If there was not a large enough sample size to perform a chi-square analysis a Fisher's Exact test was used. The following formula was used to calculate the odds ratio:

$$\begin{array}{l} Odds\;Ratio=\frac{\left(\#\;affected\;for\;breed\right)\left(\#\;normal\;mixed\;breed\right)}{\left(\#\;normal\;breed\right)\left(\#\;affected\;mixed\;breed\right)} \end{array}$$

### Pedigree Evaluation

A 3–5 generation pedigree was obtained from owners and breeders for affected and unaffected dogs when possible. This is a routine request for patients diagnosed with congenital heart disease by the UCD VMTH Cardiology service due to the ongoing genetic research initiatives of the unit (IACUC #18106 and 20047 developing genetic library). Disease status was determined based on echocardiogram by a board-certified cardiologist or cardiology resident under the supervision of a cardiologist at UCD VMTH or based on Orthopedic Foundation for Animals reported cardiac screening status determined by a board-certified cardiologist. Pedigrees were generated manually using Adobe Illustrator for SAS-affected breeds including the Bullmastiff, Golden Retriever, and Rottweiler, as well as PS-affected Bulldogs.

The following defining features were utilized to support proposal of a possible pattern of inheritance (20):

- *X-linked recessive*: There is a sex predisposition with males being affected more frequently than females. Pedigree analysis highlights females passing down the condition to almost exclusively male offspring. Affected males are unable to pass down the condition to male offspring.
- *X-linked dominant:* Pedigree analysis reveals no sex predisposition for acquiring the disease. An affected male transmits the disease to all his daughters, but no sons. Every affected individual must have an affected parent resulting in no skipping of generations.
- *Autosomal dominant*: Pedigree analysis reveals no sex predisposition for acquiring or transmitting the disease. Every affected individual must have an affected parent resulting in no skipping of generations.
- *Autosomal recessive*: Pedigree analysis reveals no sex predisposition for acquiring or transmitting the disease. An affected individual can have affected or unaffected parents. This type of inheritance may skip generations and is likely to be seen with consanguineous mating.

## Results

### Prevalence

During the period of January 2008 to December 2017, the UCD VMTH saw a total of 80,943 unique dogs between all services, and the cardiology service saw a total of 5,548 unique dogs. There was a total of 259 unique SAS and 336 unique PS cases diagnosed during this 10-year period (Table 1). No equivocal diagnoses were included in these figures and tables except when noted in the pedigrees. Furthermore, 16 dogs with PS were concurrently diagnosed with a coronary artery anomaly: 1 mixed-breed, 1 Boston Terrier, 1 Pitbull, and 13 Bulldogs. There were 80 reported SAS-affected dogs and 18 PS-affected dogs excluded based on equivocal outflow tract velocities (Supplemental Tables 1, 2). The breeds with the highest number of dogs with equivocal AoV were the Boxer (*n* = 15), Golden Retriever (*n* = 14), and mix-breed (*n* = 19) dogs. The breed with the highest number of dogs with equivocal PoV was Bulldogs (*n* = 10). All other PS-affected breeds had either one or no dogs with an equivocal PoV. The prevalence of SAS was 0.3% overall in the population and 4.7% of dogs seen by the cardiology service. The prevalence for PS was 0.41% overall in the population and 6.1% of dogs seen by the cardiology service.

**Table 1:** Number of cases diagnosed with subvalvular aortic stenosis (SAS) or pulmonic stenosis (PS) based on continuous-wave doppler echocardiogram assessment.

**Severity of disease**	**SAS**	**PS**
Mild	146	35
Moderate	33	54
Severe	80	247
Total	259	336

**Figure 2:**
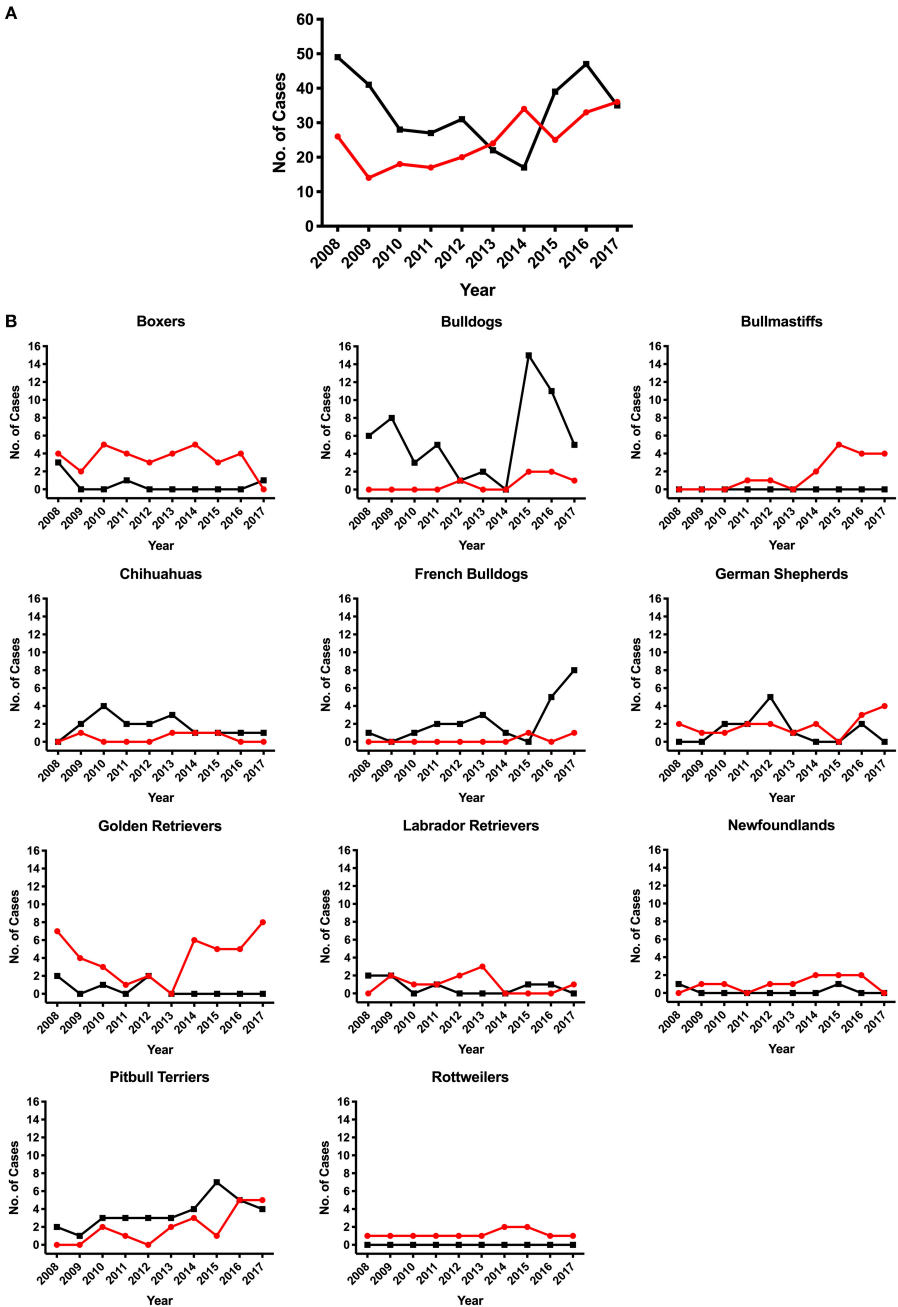
Number of subvalvular aortic stenosis (SAS) or pulmonic stenosis (PS) cases diagnosed between January 2008 to December 2017. Dogs diagnosed with SAS or PS by the cardiology service team at a large university veterinary teaching hospital. **(A)** Overall number of dogs diagnosed with SAS (red line) or PS (black line). **(B)** Breeds that contained ≥10 cases of either disease during the study period. The red line illustrates SAS cases and black line illustrates PS cases.

SAS was observed in 47 different breeds and PS was observed in 65 different breeds excluding mix-breed dogs. Overall, we observed an increase in the number of cases diagnosed with SAS at the UCD VMTH during this 10-year period (Figure 2A). The number of PS cases decreased steadily until 2014, then began increasing. Breed specific prevalence and odds ratios were calculated for breeds with ≥10 cases (Tables 2, 3). Bullmastiffs and Newfoundlands had the greatest prevalence (6.59 and 4.46%, respectively) and odds ratio (52.43 and 34.73, respectively) for SAS. We observed an increased number of Bullmastiffs, Golden Retrievers, and Pitbull Terriers diagnosed with SAS during this time frame (Figure 2B). Bulldogs and French Bulldogs were the breeds with the greatest prevalence (4.8 and 2.7%, respectively) and odds ratio (13.32 and 7.52, respectively) for PS. We observed an increased number of Bulldogs, French Bulldogs, and Pitbull Terriers diagnosed with PS during this time frame (Figure 2B).

**Table 2:** Odd ratio by breed with ≥10 cases for subvalvular aortic stenosis (SAS).

**Breed**	**No. of cases**	**Prevalence (%)**	**Odd ratio**	**95% Confidence interval**	***p*-value**
Bullmastiff	17	6.59	52.43	28.53–96.34	<0.0001
Newfoundland	10	4.46	34.73	16.77–71.95	<0.0001
Boxer	34	2.27	17.23	10.51–28.23	<0.0001
Golden retriever	42	1.42	10.67	6.67–17.08	<0.0001
Rottweiler	12	1.17	8.78	4.48–17.20	<0.0001
German shepherd	18	0.64	4.79	2.66–8.60	<0.0001
Pitbull terrier	17	0.61	4.59	2.53–8.34	<0.0001
Labrador retriever	10	0.15	1.09	0.53–2.22	0.8204
Mixed breed	30	0.13	n/a	n/a	n/a

**Table 3:** Odds ratio by breed with ≥10 cases for pulmonic stenosis (PS).

**Breed**	**No. of cases**	**Prevalence (%)**	**Odd ratio**	**95% Confidence interval**	***p*-value**
Bulldog	56	4.80	13.32	9.42–18.84	<0.0001
French bulldog	23	2.70	7.52	4.71–12.02	<0.0001
Pitbull terrier	35	1.30	3.53	2.37–5.26	<0.0001
Chihuahua	17	0.52	1.45	0.86–2.45	0.1677
German shepherd	12	0.43	1.19	0.65–2.19	0.5682
Mixed breed	80	0.38	n/a	n/a	n/a

There was 125 male and 134 female SAS cases overall. When broken down by breed, male Rottweilers had an odds ratio of 5.515 of having SAS (95% confidence interval: 1.292–25.270, *p* = 0.018). No other breed that contained ≥10 cases had a male or female predisposition (*p* < 0.05) for SAS (Supplemental Table 3). There was 192 males and 144 female PS cases overall. Male mixed-breed dogs had an odds ratio of 1.789 of developing PS when compared to females (95% confidence interval: 1.147–2.819, *p* = 0.013). No other breed that contained ≥10 cases had a male or female predisposition (*p* < 0.05) for PS (Supplemental Table 4).

### Mode of Inheritance for SAS and PS

**Figure 3:**
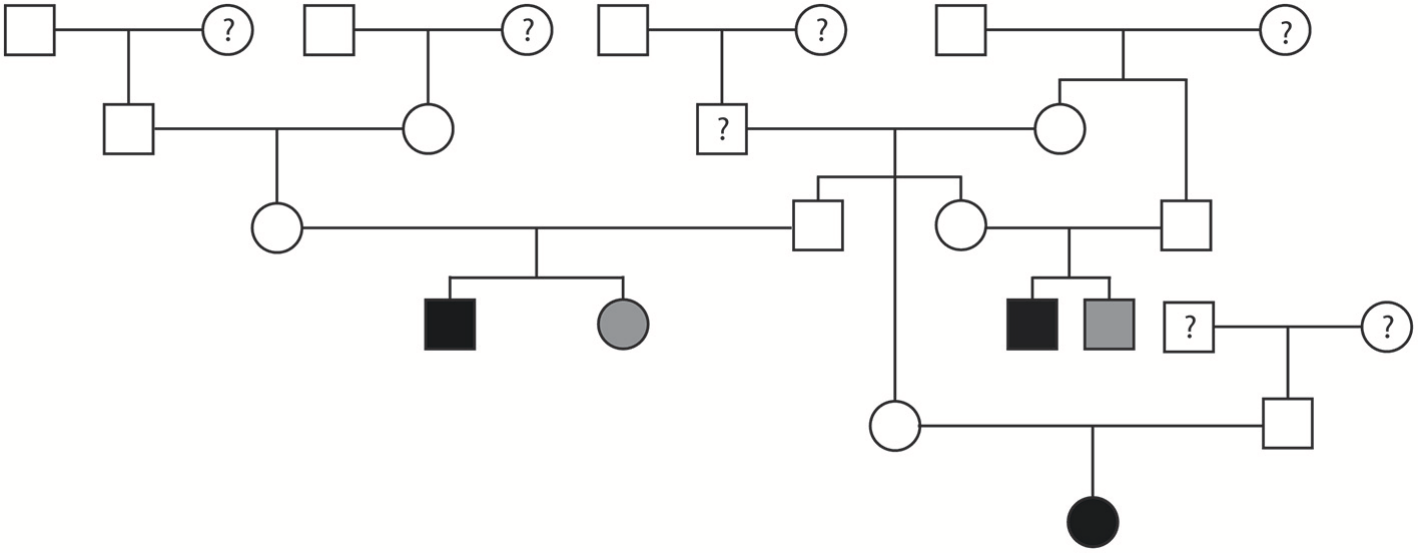
Pedigree representing an extended family of 25 Bullmastiffs phenotyped for subvalvular aortic stenosis (SAS). Affection status is represented as follows: squares represent males, circles represent females, open symbols are SAS unaffected, gray symbols are SAS equivocal, black symbols are SAS affected, and symbols with questions marks have unknown SAS cardiac status.

Pedigree evaluation was performed for a family of 25 Bullmastiffs that consisted of 13 males and 12 females (Figure 3). Two of the males (AoV = 2.6 and 2.8 m/s) and one female (AoV = 4.4 m/s) were diagnosed as affected with SAS. Additionally, both of the affected males had a direct littermate diagnosed as equivocal for SAS. The male equivocal dog had an AoV = 2.3 m/s and the female equivocal dog had an AoV = 2.2 m/s. A total of 8 dogs were considered to be unaffected via echocardiogram and had an AoV ≤ 2.0 m/s and five dogs were cleared via auscultation by a board-certified cardiologist. We did not have sufficient clinical information for seven dogs in this pedigree. The pedigree illustrates two unaffected-to-unaffected mating producing an affected and equivocal offspring and another unaffected-to-unaffected mating producing one affected female. We did not have additional information for direct littermates of the affected female dog. Furthermore, there were no affected-to-unaffected, equivocal-to-unaffected, or affected-to-equivocal mating in this pedigree. Pedigree analysis results therefore support an autosomal recessive mode of inheritance for Bullmastiffs affected with SAS.

**Figure 4:**
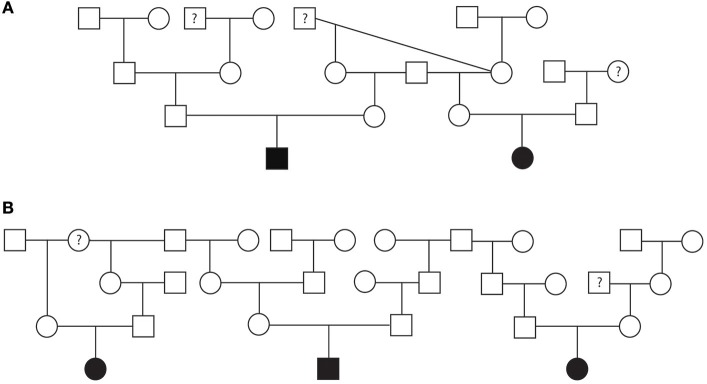
Pedigree representing two families of Golden Retrievers that were phenotyped for subvalvular aortic stenosis (SAS). **(A)** Represents first family. **(B)** Represents the second family. Affection status is represented as follows: squares represent males, circles represent females, open symbols are SAS unaffected, gray symbols are SAS equivocal, black symbols are SAS affected, and symbols with questions marks have unknown SAS cardiac status.

Pedigrees for two families of Golden Retrievers were evaluated (Figures 4A,B). The first family contained a total of 20 dogs (10 males and 10 females) and the second family contained a total of 30 dogs (14 males and 16 females). Between both families, there was a total of five Golden Retrievers diagnosed with SAS, two males and three females, with a median AoV of 4.1 m/s (range 2.6–7.0 m/s). With regards to the first family all 15 unaffected Golden Retrievers were cleared of SAS via auscultation by a board-certified cardiologist. In the second family, three Golden Retrievers were unaffected by echocardiogram (AoV ≤ 2.0 m/s) and the remaining 22 dogs were cleared of SAS via auscultation by a board-certified cardiologist. We did not have sufficient cardiac health information for three dogs in family one and two dogs in the second family. Additionally, we did not have sufficient genealogical information and health status information for direct littermates of the affected dogs. Both of these pedigrees highlight a total of five unaffected-to-unaffected matings that produced an affected offspring. There were no affected-to-equivocal matings or affected-to-affected matings in this pedigree. Pedigree analysis results for both of these Golden Retriever families support an autosomal recessive mode of inheritance for SAS affected dogs.

**Figure 5:**
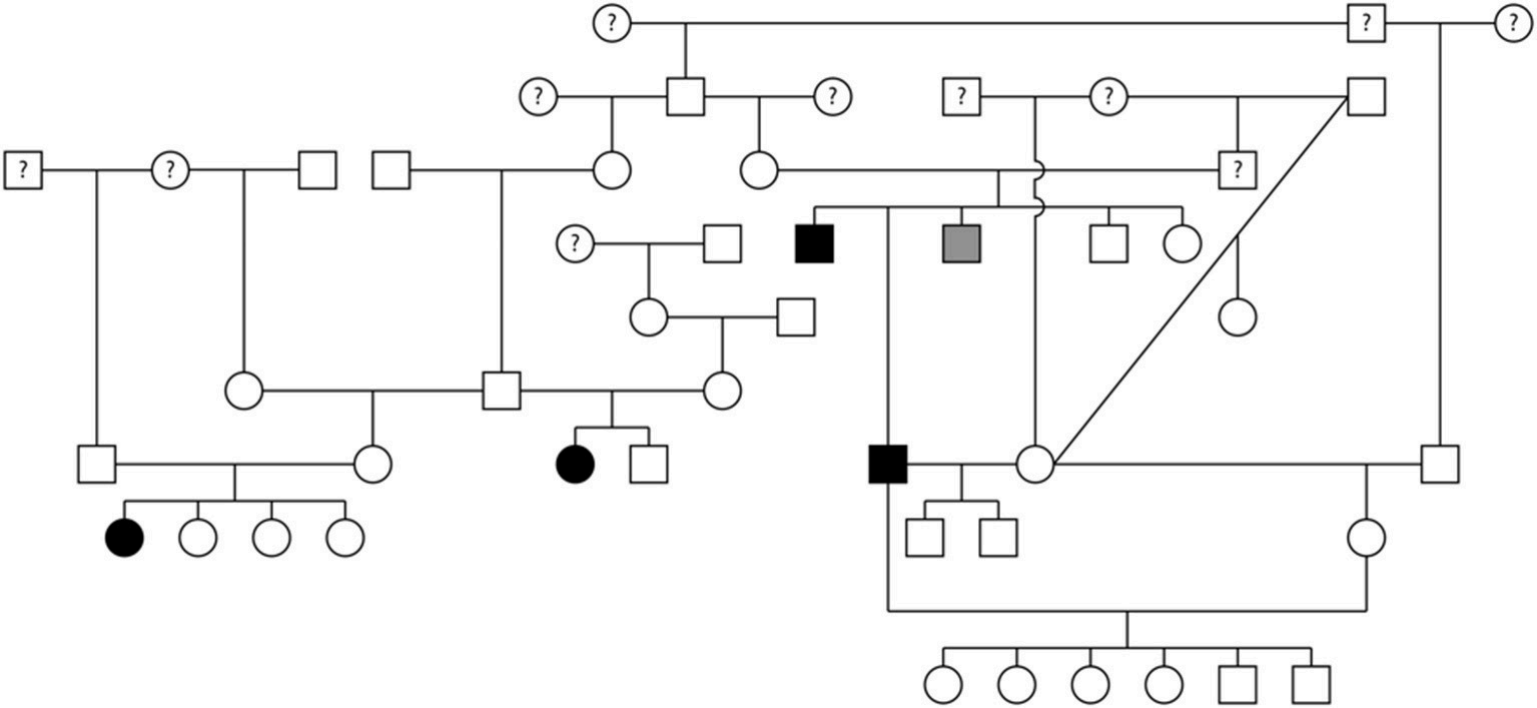
Pedigree representing an extended family of 48 Rottweilers phenotyped for subvalvular aortic stenosis (SAS). Affection status is represented as follows: squares represent males, circles represent females, open symbols are SAS unaffected, gray symbols are SAS equivocal, black symbols are SAS affected, and symbols with questions marks have unknown SAS cardiac status.

Pedigree assessment was also completed for a family of 48 Rottweilers that included 22 males and 26 females (Figure 5). Four of the Rottweilers were diagnosed with SAS, two males (AoV = 3.0 and 3.4 m/s) and two females (AoV = 3.0 and 4.9 m/s). Additionally, one male Rottweiler was diagnosed with equivocal SAS (AoV = 1.7 m/s). Although the equivocal dog had a normal AoV, 2D echocardiogram assessment indicated mild discrete narrowing of the LVOT below the aortic valve therefore was labeled as equivocally affected. Sixteen dogs were cleared of SAS via echocardiogram (AoV < 2.0 m/s) and 16 dogs were cleared via auscultation by a board-certified veterinary cardiologist. Cardiac health information was unavailable for 11 dogs included in this analysis. This pedigree highlights two unaffected-to-unaffected matings that produced both affected and unaffected offspring. Additionally, an unaffected-to-unknown mating produced affected, equivocal, and unaffected offspring. Furthermore, breeding an affected-to-unaffected Rottweiler twice produced unaffected offspring both times. There were no affected-to-affected or equivocal-to-unaffected matings reported in this pedigree. Pedigree analysis results for SAS affected Rottweilers supports an autosomal recessive mode of inheritance.

**Figure 6:**
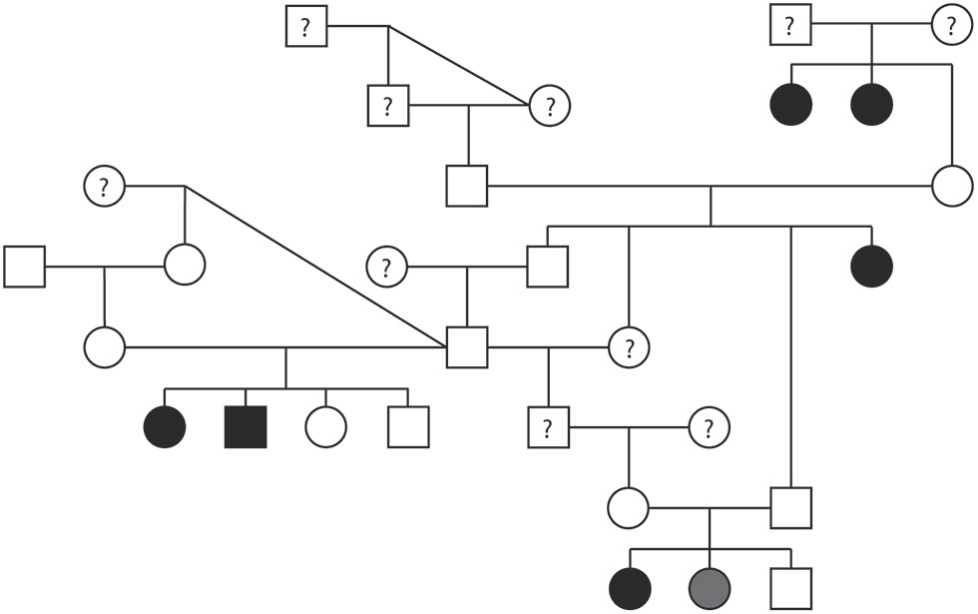
Pedigree representing an extended family of 29 Bulldogs phenotyped for pulmonic stenosis (PS). Affection status is represented as follows: squares represent males, circles represent females, open symbols are PS unaffected, gray symbols are PS equivocal, black symbols are PS affected, and symbols with questions marks have unknown PS cardiac status.

A pedigree evaluation was performed in a family of 29 Bulldogs that contained 12 males and 17 females (Figure 6). There was a total of six Bulldogs diagnosed with PS (5 females and 1 male) and one equivocal female. The PoV of the affected dogs ranged from 3.8 to 6.07 m/s. The equivocal female had a PoV of 2.0 m/s. Eight dogs were cleared of PS via echocardiogram (PoV < 1.5 m/s) and four dogs were cleared via auscultation by a board-certified cardiologist. Cardiac health information was unavailable for ten dogs included in this pedigree. There were three matings of unaffected parents which resulted in a litter with one or more PS-affected offspring. There were no affected-to-affected, affected-to-unaffected, or equivocal-to-unaffected matings reported in this pedigree. There were several consanguineous matings present. These findings support an autosomal recessive mode of inheritance for PS in Bulldogs.

## Discussion

### Prevalence

For this retrospective study, we analyzed the case records from the UCD VMTH to determine the number of dogs diagnosed with either SAS or PS. The prevalence of SAS and PS obtained in this study (4.7 and 6.1%, respectively) are higher than what was reported in previous publications (1, 2, 21, 22). This could be due to the fact that UCD VMTH is a referral hospital, therefore cases are referred here that are identified elsewhere. Another explanation is that the cardiologists at UC Davis offer multiple clinical trials and ongoing genetic studies that may impact the number of dogs referred to this institution and may at least in part account for the increases in SAS cases in 2014 and PS cases in 2015. These types of confounding factors are frequently identified in prevalence studies from referral institutions which is largely where the current statistics on congenital heart disease prevalence are generated.

SAS was observed in 47 different breeds and PS was observed in 65 breeds excluding mix-breed dogs. Not only are these diseases widespread across dogs, but they also afflict the five most popular breeds according to the 2017 AKC rankings^1^. The breeds identified in this study as being predisposed to SAS have also been reported previously with the exception of Bullmastiffs and Pitbull Terriers (2, 7, 22). Golden Retrievers had the highest number of cases, while Bullmastiffs had the highest odds ratio. The increase in reported SAS cases in Bullmastiffs can be attributed to improved cardiac screening practices that began being recommended by the breed club in 2014. This highlights the importance of echocardiogram clearances for breeds with a high predisposition for SAS, as cardiac auscultation-only clearance can miss mildly affected cases.

The breeds identified in this study as being predisposed to PS have also been reported in the previous literature (2, 7, 22). It is well-known that Bulldogs are predisposed to both PS and coronary anomalies (2, 9). In this study, Bulldogs had the most cases of PS, highest odds ratio for PS, and the highest number of coronary anomalies. Although French Bulldogs were predisposed to PS, there were no French Bulldogs diagnosed with a coronary anomaly via angiography which is consistent with other studies (23). Although Boxers had a high prevalence of PS in previous literature (2, 9), there were very few cases in Boxer dogs seen during the time period studied. A similar temporal trend was demonstrated in Italy following the introduction of stringent screening practices for the Boxer breed (24). The American Boxer Club implemented strict cardiac screening practices in 2000^2^, which could explain the reduction in cases since affected dogs have been discouraged from breeding.

In a mixed-breed shelter population PS was the most prevalent congenital heart disease, while SAS was the 3rd most common (1). In the mixed-breed dogs in this study, 30 were affected with SAS and 80 with PS. Interestingly, mixed-breeds are commonly mentioned as having high prevalence of PS in many studies (2, 9, 25), indicating that PS is not simply a disease of purebred dogs.

There was an interesting discrepancy between the severity of disease in SAS and PS cases with the majority of SAS cases being mild, while the majority of PS cases were severe. This is likely due to the study population being at a referral hospital and thus not representative of the severity distribution of disease in the general population. For example, the majority of dogs diagnosed with SAS at this hospital occur due to evaluation of a left basilar systolic heart murmur detected prior to a referral procedure unrelated to the heart. Since surgical intervention is not common or recommended for SAS cases, it is suspected that the majority of SAS cases are managed medically by board-certified cardiologists outside of UCD VMTH. For PS, moderate and severe cases are often referred to UCD VMTH specifically for a balloon valvuloplasty surgery, while mild and many moderate PS cases do not require intervention and are thus likely not referred for evaluation. The remaining equivocal or mild PS cases were diagnosed after a murmur was detected during a routine physical exam or prior to a referral procedure unrelated to the heart. It is important to note that for both PS and SAS many of these mild cases were not displaying clinical signs at the time of diagnosis, thus stressing the importance of stringent cardiac screening practices prior to breeding in at-risk dog breeds.

### Mode of Inheritance

None of the breeds with more than 10 cases had a statistically significant difference in the number of males and females except for SAS Rottweilers and PS mixed-breeds. However, the male predisposition identified in the Rottweilers and mixed-breeds is likely not genetically significant. For Rottweilers even though males have a greater odds of developing SAS, X-linked inheritance is unlikely given that unaffected males produced affected female offspring.

A pedigree for SAS affected Bullmastiffs, Golden Retrievers, and Rottweilers was generated to determine the mode of inheritance for each of these breeds. To our knowledge, this is the first reported SAS pedigree evaluation for the Bullmastiff breed. The pedigree suggests that SAS in Bullmastiffs is inherited as an autosomal recessive trait. For the Golden Retrievers, a previous study suggested that SAS can be inherited as an autosomal recessive or polygenic trait (16). Our pedigree evaluation in this study further supports Stern et al. findings. Results for the Rottweiler pedigree also suggest an autosomal recessive mode of inheritance in this breed.

A pedigree for PS affected Bulldogs also suggested an autosomal recessive mode of inheritance. Interestingly, there was variable expressivity for the disease, since litters with multiple affected puppies had different severities. Both the recessive mode of inheritance and variable expressivity supports what was previously found in Beagles (5).

One limitation of the SAS and PS pedigrees is that some control samples were not evaluated via echocardiogram by a board-certified cardiologist and we only had results from their auscultation exams. Therefore, we advise readers to take this into consideration since relatives of PS or SAS affected dogs that were cleared by auscultation are sometimes diagnosed with either equivocal or mild disease by echocardiogram.

### Limitations

One of the limitations in this study is that we sampled from a single hospital population, which may not be representative of the true canine population. While larger, multicenter studies are ideal, they also add variability in diagnostic methodologies and frequently have limited data on pedigrees or patterns of inheritance. Additionally, this was a retrospective study with a lack of follow-up evaluations. Although SAS and PS have a standard echocardiographic diagnosis (6), these cases were diagnosed by different cardiologists over the 10-year period. This can lead to measurement discrepancies between cardiologists. For example, the velocity values used to determine normal, equivocal, and mild PS and SAS cases varies in the literature. This study used a higher velocity cutoff for the equivocal range which would have reduced the number of cases identified leading to a prevalence underestimation. Furthermore, our prevalence calculations can be underestimated since not all breeds require echocardiogram cardiac clearances. This may lead to a higher number of auscultation clearances which cannot definitively determine whether a dog is cleared of SAS and/or PS.

There are several other reasons why our prevalence estimates may be underestimated. We only included dogs that received an echocardiogram from a board-certified cardiologist. A murmur may be difficult to detect in certain breeds such as those with brachycephaly which may result in those dogs not being referred to a cardiologist. Therefore, some of those cases might be missing from the affected population. Finally, we only included dogs that were diagnosed antemortem by echocardiography. Therefore, prevalence calculations can be underestimated by not including stillborn or neonatal deaths (26). Despite all these limitations, this retrospective study identified a higher prevalence than previously reported and warrants consideration that these defects may be more frequent than previously thought or perhaps increasing in recent years.

## Conclusion

This study demonstrated that PS was at a higher prevalence than SAS in a veterinary hospital population from 2008–2017. Both diseases have increased in this hospital population in recent years when compared to previous literature. For SAS affected Bullmastiffs, Golden Retrievers, and Rottweilers, pedigree analysis suggests an autosomal recessive pattern of inheritance. The mode of inheritance for PS in the most affected breed, Bulldogs, also appears to be autosomal recessive. This has important implications for breeding practices and emphasizes the ultimate need for a reliable genetic screening test.

## Ethics Statement

This study was carried out in accordance with the recommendations of University of California Davis Institution of Animal Care and Use Committee. The protocol was approved by the University of California Davis Institution of Animal Care and Use Committee IACUC #18106 and 20047.

## Author Contributions

EO, SF, and JS contributed to conception and design of the study. EO and SF collected the data and performed the statistical analysis. TH, CG-H, LV contributed to phenotyping and collection of pedigree information. EO, SF, and JS wrote the original manuscript. AC generated a portion of the figures. All authors contributed to manuscript revision, read, and approved the final submitted version.

### Conflict of Interest Statement

The authors declare that the research was conducted in the absence of any commercial or financial relationships that could be construed as a potential conflict of interest.

## Footnotes

^1^AKC Staff. *Most Popular Dog Breeds—Full Ranking List*. American Kennel Club. Available online at: https://www.akc.org/expert-advice/news/most-popular-dog-breeds-full-ranking-list/ (Accessed March 28, 2018).

^2^Wallnew W. *Boxer Cardiomyopathy*. American Boxer Club (2000). Available online at: https://americanboxerclub.org/PDF/Wallner_2000.pdf

## Abbreviations

PS: Pulmonic stenosis
SAS: Subaortic stenosis
UCD VMTH: University of California-Davis Veterinary Medical Teaching Hospital
AoV: Aortic outflow velocity
PoV: Pulmonic outflow velocity.
