# Supplementary material for: Congenital Cardiac Outflow Tract Abnormalities in Dogs: Prevalence and Pattern of Inheritance From 2008 to 2017
Source: Front Vet Sci. 2019 Feb 27;6:52. doi: 10.3389/fvets.2019.00052 (PMC6402372; doi:10.3389/fvets.2019.00052)
Supplement: Supplementary file 1 [file Data_Sheet_1.docx]

**Supplemental Table 1: Number of equivocal dogs affected with SAS**

| **Breed** | **No. of Equivocal Dogs** |
| --- | --- |
| Afghan Hound | 2 |
| Australian Cattle Dog | 1 |
| Australian Shepherd | 1 |
| Bouvier des Flandres | 1 |
| Boxer | 15 |
| Bull Terrier | 5 |
| Bulldog | 2 |
| Dogo Argentino | 1 |
| German Shepherd | 1 |
| Golden Retriever | 14 |
| Labrador Retriever | 2 |
| Miniature Pinscher | 2 |
| Mix Breed | 19 |
| Newfoundland | 4 |
| Pit Bull Terrier | 2 |
| Rhodesian Ridgeback | 1 |
| Rottweiler | 4 |
| Miniature Schnauzer | 1 |
| Weimaraner | 1 |
| Whippet | 1 |

**Supplemental Table 2: Number of equivocal dogs affected with PS**

| **Breed** | **No. of Equivocal Dogs** |
| --- | --- |
| Boxer | 1 |
| Bulldog | 10 |
| Bullmastiff | 1 |
| Bull Terrier | 1 |
| Cavalier King Charles Spaniel | 1 |
| Miniature Schnauzer | 1 |
| Mixed Breed | 1 |
| Newfoundland | 1 |
| Shiba Inu | 1 |

| **Breed** | **Males** | **Females** | **Odd Ratio** | **95% Confidence Interval** | **P-values** |
| --- | --- | --- | --- | --- | --- |
| Boxer | 18 | 16 | 0.996 | 0.504 to 1.947 | >0.999 |
| Bullmastiff | 9 | 8 | 0.891 | 0.358 to 2.363 | 0.808 |
| German Shepherd | 12 | 6 | 1.671 | 0.663 to 4.318 | 0.349 |
| Golden Retrievers | 20 | 22 | 0.794 | 0.431 to 1.434 | 0.534 |
| Labrador Retrievers | 2 | 8 | 0.234 | 0.050 to 0.938 | 0.058 |
| Newfoundland | 3 | 7 | 0.457 | 0.126 to 1.705 | 0.339 |
| Pitbull Terrier | 6 | 11 | 0.500 | 0.189 to 1.324 | 0.223 |
| Rottweiler | 10 | 2 | 5.515 | 1.292 to 25.270 | 0.018 |
| Mixed Breed | 10 | 20 | 0.480 | 0.234 to 1.028 | 0.067 |

**Supplemental Table 3: Number of males and females for breeds with >10 cases for subvalvular aortic stenosis (SAS).** Odd ratio’s, 95% confidence intervals, and corresponding p-values are listed.

**Supplemental Table 4: Number of males and females for breeds with >10 cases for pulmonic stenosis (PS).** Odd ratio’s, 95% confidence intervals, and corresponding p-values are listed.

| **Breed** | **Males** | **Females** | **Odd Ratio** | **95% Confidence Interval** | **P-values** |
| --- | --- | --- | --- | --- | --- |
| Bulldog | 34 | 22 | 1.139 | 0.670 to 1.982 | 0.680 |
| Chihuahua | 4 | 13 | 0.335 | 0.119 to 0.972 | 0.052 |
| French Bulldog | 15 | 8 | 1.410 | 0.617 to 3.179 | 0.524 |
| German Shepherd | 6 | 6 | 0.834 | 0.266 to 2.615 | 0.779 |
| Pitbull Terrier | 19 | 22 | 0.792 | 0.419 to 1.450 | 0.530 |
| Mixed Breed | 52 | 28 | 1.789 | 1.147 to 2.810 | 0.013 |
